# Foreign Summary

**Published:** 1838-08-29

**Authors:** 


### FOREIGN SUMMARY.

*On the Use of the Subcarbonate of Iron (Ferri Sesquioxydum,) in Hooping-Cough. By* Dr. Steymann.

Of all the medicines recommended in this painful and lingering disease, the subcarbonate of iron, according to Dr. Steymann, when exhibited in the second stage, is by far the best.

The following cases prove the utility of this remedy.

Case I. Henri Schrœder, eleven years old, had been suffering for nine weeks from hooping-cough, for which all the usual remedies had been exhibited, without relief. The subcarbonate of iron was administered in the dose of two grains every three hours. After the tenth dose of this remedy, all the symptoms were considerably diminished. Ten doses more, each consisting of five grains, completely subdued the disease.

Case II. The sister of the former patient, five years old, was affected with the cough at the same period, had been treated in the same way, and with the same results. She commenced with two grains of the subcarbonate every three hours; this was increased to three grains, which completed the cure in about eight days. From the first dose the symptoms were alleviated.

Case III. Jules Etier, five years old, was placed under the care of Dr. S. in the third week of the disease. The cough did not yield till she took the iron; and, after four days' treatment, she was completely cured. Dr. S. says he never heard the fits of coughing more violent than in this child.

Dr. S. holds out a caution against the exhibition of this remedy in the early stages, as he has found it at this period produce considerable irritation, in lieu of hastening the cure. In the first stage he employs leeches, opiates, and emetics; and, before commencing the subcarbonate, he recommends the exhibition of an emetic.

The following he has found a convenient formula: Subcarbonate of iron, twenty-five grains, white sugar, a sufficient quantity to make into ten powders; one to be taken every three hours. This dose is increased according to the age of the patient, adding a grain for every year. The effect is generally prompt; and in a few days nothing remaining but a slight catarrhal cough, which gradually disappears.—*Bulletin Général de Therapeutique, from Brit. and For. Med. Rev.*, July, 1838.

It would appear from a letter to the editor of the American Journal of the Medical Sciences, published in the last number of that Journal, that Dr. James H. Dickson, of New York, was the first to perform Stromeyer's operation for club-foot, in America, he having operated successfully in January, 1835, whilst living in Fayetteville, N. C. Professor N. P. Smith, of Lexington, also performed this operation with complete success in 1836, and in 1837 with a like result.

In this city it has been performed by Drs. G. W. Norris and Mutter. Their cases are doing well.

